# DUS Topp–Leone-G Family of Distributions: Baseline Extension, Properties, Estimation, Simulation and Useful Applications

**DOI:** 10.3390/e26110973

**Published:** 2024-11-13

**Authors:** Divine-Favour N. Ekemezie, Kizito E. Anyiam, Mohammed Kayid, Oluwafemi Samson Balogun, Okechukwu J. Obulezi

**Affiliations:** 1 Department of Statistics, Faculty of Physical Sciences, Nnamdi Azikiwe University, P.O. Box 5025, Awka 420007, Nigeria; de.ngozi@stu.unizik.edu.ng; 2 Department of Statistics, College of Physical Sciences, Federal University of Technology, Owerri 460114, Nigeria; kizito.anyiam@futo.edu.ng; 3 Department of Statistics and Operations Research, College of Science, King Saud University, P.O. Box 2455, Riyadh 11451, Saudi Arabia; bdrkayid@ksu.edu.sa; 4 Department of Computing, University of Eastern Finland, FI-70211 Kuopio, Finland; samsb@student.uef.fi

**Keywords:** Dinesh–Umesh–Sanjay transformer, estimation, exponential distribution, HIV/AIDS mortality rate, Topp–Leone family, total factor productivity

## Abstract

This study introduces the DUS Topp–Leone family of distributions, a novel extension of the Topp–Leone distribution enhanced by the DUS transformer. We derive the cumulative distribution function (CDF) and probability density function (PDF), demonstrating the distribution’s flexibility in modeling various lifetime phenomena. The DUS-TL exponential distribution was studied as a sub-model, with analytical and graphical evidence revealing that it exhibits a unique unimodal shape, along with fat-tail characteristics, making it suitable for time-to-event data analysis. We evaluate parameter estimation methods, revealing that non-Bayesian approaches, particularly Maximum Likelihood and Least Squares, outperform Bayesian techniques in terms of bias and root mean square error. Additionally, the distribution effectively models datasets with varying skewness and kurtosis values, as illustrated by its application to total factor productivity data across African countries and the mortality rate of people who injected drugs. Overall, the DUS Topp–Leone family represents a significant advancement in statistical modeling, offering robust tools for researchers in diverse fields.

## 1. Introduction

In applied statistics and probability theory, the development of new probability distributions plays an essential role in effectively modeling complex, real-world phenomena. Traditional distributions, while powerful in many contexts, can sometimes fall short in capturing the full range of variability and specific patterns inherent in certain types of data. This is especially true in fields where the observed data exhibit unique skewness, kurtosis, or heavy tails that require a more flexible model to yield accurate insights. One of the key areas where advanced probability distributions are particularly valuable is in the analysis of lifetime events. These events, such as the time until failure of a machine part or the duration until recovery from a disease, often show complex patterns that cannot be fully represented by conventional distributions like the exponential or normal. In this study, we introduce a new family of probability distributions and an extended form of the exponential distribution specifically designed to capture the nuances of lifetime data. This new distribution has potential applications across various domains, offering a fresh approach to model events over time and providing more nuanced interpretations of underlying trends. One significant application of advanced probability distributions is in economics, particularly in assessing total factor productivity (TFP) across countries. TFP is a critical indicator of economic efficiency and is integral to understanding the growth trajectories of nations. By modeling the variability in TFP using a tailored probability distribution, researchers can uncover factors that drive productivity, which are not always visible in standard economic analyses. Such insights can illuminate the role of technological progress, policy changes, and labor productivity, providing policymakers with valuable information to shape strategies for sustainable development. For instance, examining the distributional characteristics of TFP may reveal how certain economies are prone to stagnation, while others exhibit robust growth resilience, despite similar inputs. This type of analysis has profound implications for development economists, who strive to understand and enhance the determinants of economic progress on a global scale. In public health, accurately modeling mortality trends is paramount, especially in the context of chronic conditions like HIV/AIDS. The global impact of HIV/AIDS on mortality rates remains substantial, affecting millions of lives and posing a persistent public health challenge. Using advanced probability distributions to model HIV/AIDS mortality data enables public health officials and researchers to gain deeper insights into trends over time, variations across demographic groups, and potential factors that influence mortality. For example, applying the proposed probability distribution can help to identify shifts in mortality patterns due to advancements in antiretroviral therapy (ART) or changes in socio-economic conditions. By accurately capturing these patterns, the model serves as a powerful tool for assessing the effectiveness of public health interventions, thereby providing a robust foundation for policy recommendations and future research in epidemiology.

The introduction of this new family of probability distributions extends beyond economics and public health, with applicability in engineering, environmental science, and social sciences where complex, real-world processes require more adaptable statistical models. In engineering, for instance, modeling failure times of components with this new distribution can lead to improved reliability assessments and maintenance strategies, ultimately enhancing system efficiency and safety. In environmental science, analyzing patterns in climate variables or biodiversity measures using flexible probability models can yield critical insights into ecological stability and risks posed by environmental change. This study contributes a valuable new tool to the field of applied statistics, addressing the need for more adaptable probability models to capture the subtleties of real-world data. The new family of probability distributions holds promise for improving the accuracy and interpretability of statistical models across disciplines, enriching the understanding of complex phenomena in a way that benefits both academic research and practical decision-making. Several techniques have been utilized in the development of new families of distributions to enhance existing distributions. A number of these techniques are presented in Table 1 below.

Regarding fitting probability distributions to medical and related data, this study is not in isolation. Several authors have undertaken studies in this direction, leading to a rich literature base covering applications of certain probability distributions to medical and biomedical events. For instance, Al-Noor et al. [17] presented the Marshall–Olkin–Marshall–Weibull distribution. This distribution was designed to fit into two datasets for the early detection of breast tumors in Egypt and fatigue fracture of Kevlar 373 (epoxy) subjected to a 90% stress level. This distribution was found to have performed better than the competing models based on the two datasets. Feroze et al. [18] introduced the modified Weibull extension and applied it to a medical dataset with a Bayesian perspective under different loss functions and informative priors. The Topp–Leone modified Weibull distribution proposed by Alyami et al. [19] was used to model datasets related to medical data and the results were impressive. The weighted inverse Weibull distribution with application introduced by AbaOud and Almuqrin [20] exhibited heavy tail characteristics and was used to model medical data. In addition, the bounded exponentiated Weibull distribution studied by Bashir et al. [21] considered COVID-19 survival rates in Spain as an illustration. Other studies devoted to applications to medical data include the works of Mazucheli et al. [22], Qura et al. [23], Tolba et al. [24], and El-Morshedy et al. [25].

**Figure 1:**
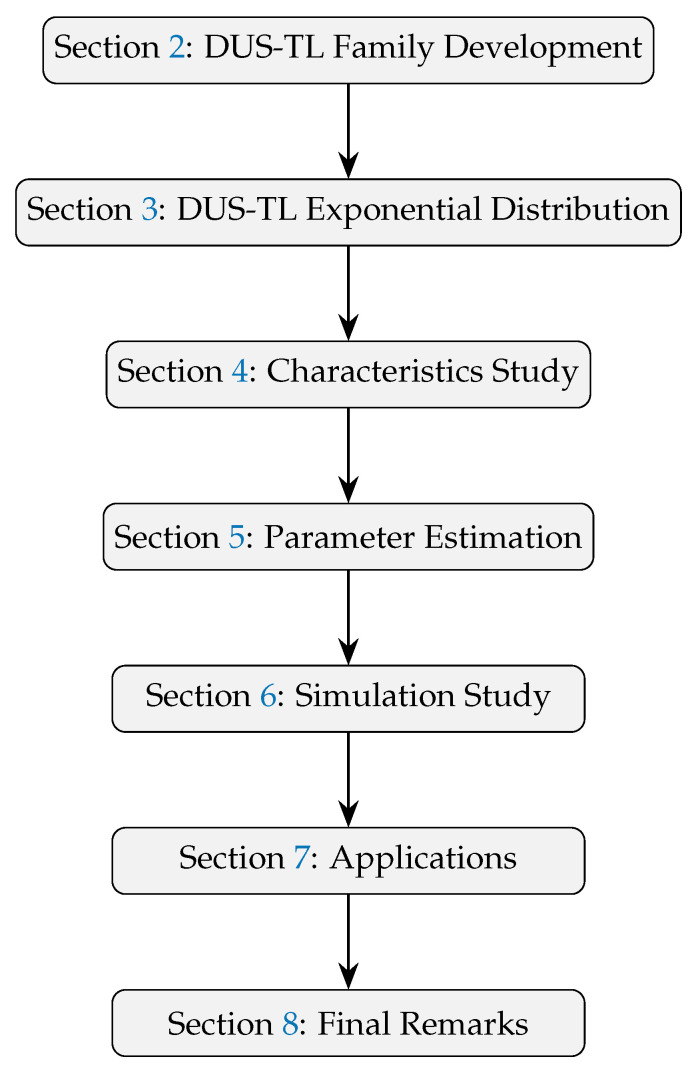
Outline of the remaining sections of this study.

The motivation for the development of the new family of distributions in this study ranges from the need to provide models that are parameter parsimonious (lower number of parameters), wider applicability of the attendant new distributions compared to the parent distribution, and improved goodness of fit, estimation adaptability and mathematical flexibility. It is easy to see that the new family formulated in Section 2 did not add any parameters to the Topp–Leone family of distributions; rather, its functional form is altered, rendering a more flexible, adaptable and tractable Topp–Leone family with a better goodness of fit. The remainder of this study is presented in Figure 1 below.

## 2. The DUS Topp–Leone Family of Distributions

Rezaei et al. [26] introduced the Topp–Leone generated family of distribution with cumulative distribution function (CDF) and probability density function (PDF) expressed respectively:

(1) $$H\left(x;\nu ,\xi \right)={\left[1-{\left(1-G(x)\right)}^{2}\right]}^{\nu },$$

and

(2) $$h\left(x;\nu ,\xi \right)=2\nu g\left(x;\xi \right)\left(1-G(x;\xi )\right){\left[1-{\left(1-G(x;\xi )\right)}^{2}\right]}^{\nu -1}.$$

Another interesting generalized generator of distributions is the Dinesh–Umesh–Sanjay (DUS), which was formulated by Kumar et al. [16] with CDF and PDF denoted as follows, respectively:

(3) $$F\left(x;\xi \right)=\frac{e^{H(x;\xi )}-1}{e-1},$$

and

(4) $$f\left(x;\xi \right)=\frac{h\left(x;\xi \right)e^{H(x;\xi )}}{e-1}.$$

If we substitute Equations (1) and (2) into Equations (3) and (4), a new generalized family of distributions named the DUS Topp–Leone family is birthed, with CDF and PDF presented as follows, respectively:

(5) $$F\left(x;\nu ,\xi \right)=\frac{e^{{\left[1-{\left(1-G(x;\xi )\right)}^{2}\right]}^{\nu }}-1}{e-1};\;x>0,\;\nu >0,$$

and

(6) $$f\left(x;\nu ,\xi \right)=\frac{2\nu g\left(x;\xi \right)\left(1-G(x;\xi )\right){\left[1-{\left(1-G(x;\xi )\right)}^{2}\right]}^{\nu -1}e^{{\left[1-{\left(1-G(x;\xi )\right)}^{2}\right]}^{\nu }}}{e-1}.$$

ξ is a vector of the parameters of the baseline distribution whose CDF and PDF are G(x;ξ) and g(x;ξ), respectively. The linear form of the PDF is obtained by introducing the power and binomial series expansion identities as follows:

f(x;ν,ξ)=2νg(x;ξ)G¯(x;ξ)e−1∑i=0∞∑j=0∞∑k=0∞∑l=0∞∑m=0∞(−1)i+k+l+mj!ν−1i2ikνjl2lmGk+m(x;ξ),

where $\bar{G}\left(x;\xi \right)=1-G\left(x;\xi \right)$.

## 3. Baseline Extension: DUS Topp–Leone Exponential (DUS-TLE) Distribution

Suffice it to say that the exponential distribution is the most popular non-negative light-tailed standard distribution, with CDF and PDF given as $G\left(x;\rho \right)=\rho e^{-\rho x}$ and $g\left(x;\rho \right)=\rho e^{-\rho x}$. Utilizing the exponential distribution by making appropriate replacements in Equations (5) and (6), we obtain the DUS-TLE distribution with CDF and PDF given as

(7) $$F\left(x;\nu ,\rho \right)=\frac{e^{{\left[1-e^{-2\rho x}\right]}^{\nu }}-1}{e-1};\;x>0,\;\nu ,\;\rho >0,$$

and

(8) $$f\left(x;\nu ,\rho \right)=\frac{2\nu \rho e^{-2\rho x}{\left[1-e^{-2\rho x}\right]}^{\nu -1}e^{{\left[1-e^{-2\rho x}\right]}^{\nu }}}{e-1}.$$

ν is the additional shape parameter from the DUS-TL family for the enhancement of any baseline distribution and ρ is the scale parameter of the exponential distribution. It is noteworthy that the embedding of the DUS transformer into the TL family only alters the functional form of the TL family. Hence, we can be sure that the attendant distribution has versatility and efficacy in modeling lifetime events across varied contexts, from economic productivity to health outcomes.

The hazard rate function (HRF), denoted as h(x;ν,ρ) and derived from the given PDF and CDF, can be written as

(9) $$h\left(x;\nu ,\rho \right)=\frac{2\nu \rho e^{-2\rho x}{\left(1-e^{-2\rho x}\right)}^{\nu -1}e^{{\left[1-e^{-2\rho x}\right]}^{\nu }}}{e-e^{{\left[1-e^{-2\rho x}\right]}^{\nu }}}.$$

**Figure 2:**
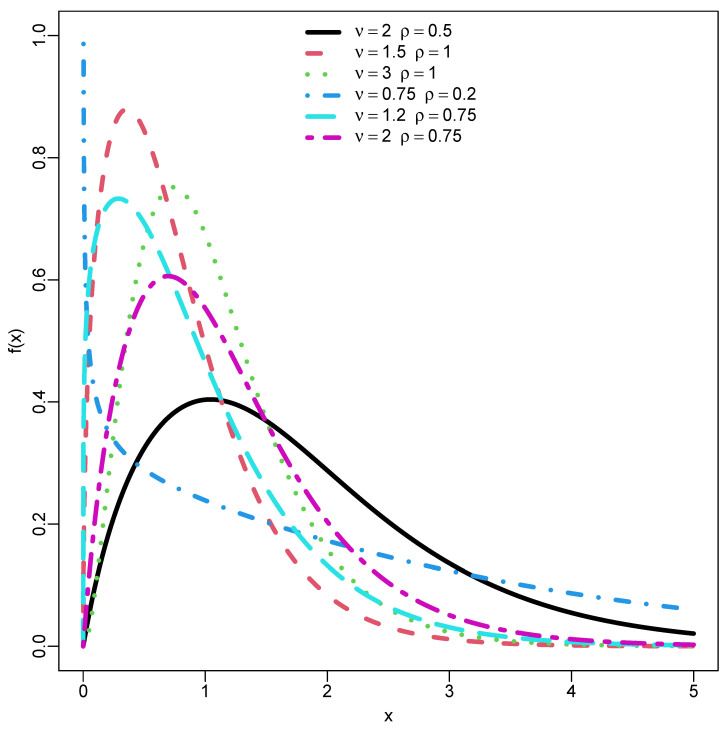
PDF plots of DUS-TLE (ν,ρ).

Figure 2 is the PDF of the suggested DUS-TLE distribution exhibiting a unimodal shape, reversed bathtub shape and fat-tail.

### 3.1. Extreme Behavior of the DUS-TLE Distribution

The PDF of the DUS-TLE distribution exhibits interesting behavior as *x* approaches both 0 and *∞*. As x→0, the term $e^{-2\rho x}$ tends to 1, and consequently, $1-e^{-2\rho x}$ approaches 0. Therefore, the function tends to 0 due to the factor ${\left(1-e^{-2\rho x}\right)}^{\nu -1}$ vanishing when ν>1. This implies that for values of ν>1, the function converges to zero at the origin.

Similarly, as x→∞, the term $e^{-2\rho x}$ decays exponentially to 0, and $1-e^{-2\rho x}\to 1$. In this limit, the function also tends to 0, as the leading factor $e^{-2\rho x}$ dominates, causing an exponential decay. The parameter ρ governs the rate at which the function decays to zero for large *x*, with higher values of ρ resulting in faster convergence.

From these observations, we can deduce that the function is bounded at both extremes, meaning it converges to zero as x→0 and as x→∞. This suggests that the function has a local maximum at some intermediate value of *x*. For typical values of ν>1 and ρ>0, the function likely exhibits a “hump”-like behavior, where it rises from 0, reaches a peak, and then decays back to 0 as *x* increases. The exact location and height of this peak depend on the parameters ν and ρ. Larger values of ν influence the shape of the peak, making it sharper or broader, while ρ affects the rate of decay as *x* increases.

To ensure the function converges appropriately, the parameter ν must be greater than 1, since for ν=1, the term ${\left(1-e^{-2\rho x}\right)}^{\nu -1}$ would not vanish as x→0, potentially altering the behavior near the origin. Additionally, ρ>0 ensures the exponential decay necessary for the function to converge to 0 as x→∞.

Overall, the function describes a bounded behavior, with convergence to 0 at both extremes, and it is likely to have a peak for intermediate values of *x*. The parameters ν and ρ play a critical role in determining the shape and rate of decay of the function, making it potentially useful for modeling phenomena where intensity rises and falls, such as life distributions or time-to-event models.

**Figure 3:**
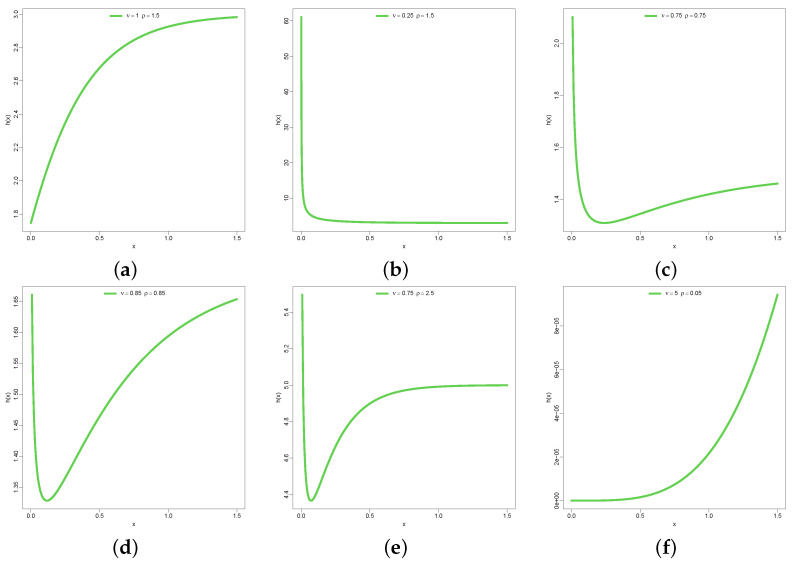
h(x) of DUS-TLE.

Figure 3a–f represent the hazard function with varying behavior or patterns of shape. Some are right-tailed, others increasing, left-tailed, bathtub and bump-like shapes. These are evidence of the versatility of the model in providing fit to lifetime data of various natures.

### 3.2. Mixture Representation

The PDF of the DUS-TLE distribution in Equation (8) can be reduced further using the power series expansion $e^{x}=\sum_{j=0}^{\infty }\frac{x^{j}}{j!}$ and the binomial series expansion (1−x)n=∑i=0∞(−1)inixi for ∣x∣<1. Therefore, we rewrite the PDF as

(10) f(x;ν,ρ)=2νρ∑i=0∞∑j=0∞∑k=0∞(−1)i+kν−1ijνke−2ρ(1+k+i)x.

## 4. Characteristics

In this section, some properties of the DUS-TLE distribution are discussed, which include the moment and its measures, moment-generating function, quantile function, entropy and order statistic.

### 4.1. Moment

The *s*-th moment of a random variable which assumes the DUS-TLE distribution can be expressed as

(11) μr′=2νρ∑i=0∞∑j=0∞∑k=0∞(−1)i+kν−1ijνk2ρ(1+k+i)−(s+1)Γ(s+1);s=1,2,⋯

The first moment which coincidentally is the mean is obtained by substituting s=1 in Equation (11), hence

μ=2νρ∑i=0∞∑j=0∞∑k=0∞(−1)i+kν−1ijνk2ρ(1+k+i)−2;sinceΓ(2)=1.

Similarly, the second, third and fourth moments are, respectively,

μ2′=4νρ∑i=0∞∑j=0∞∑k=0∞(−1)i+kν−1ijνk2ρ(1+k+i)−3;sinceΓ(3)=2,

μ3′=12νρ∑i=0∞∑j=0∞∑k=0∞(−1)i+kν−1ijνk2ρ(1+k+i)−4;sinceΓ(4)=6,

and

μ4′=48νρ∑i=0∞∑j=0∞∑k=0∞(−1)i+kν−1ijνk2ρ(1+k+i)−5;sinceΓ(5)=24.

**Figure 4:**
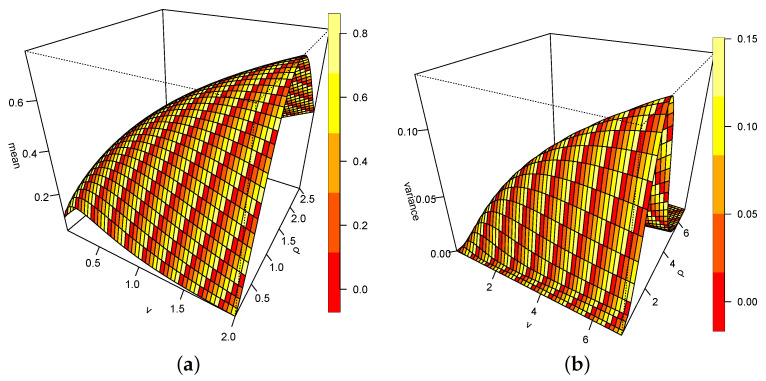
(**a**) Mean of DUS-TLE. (**b**) Variance of DUS-TLE.

Figure 4a,b represent the mean and variance in 3D graphics. A close observation reveal that for increasing values of the ν and ρ, the mean becomes large. The peak is at ρ=1.5 and ν=2. Values of ρ>1.5 cause the mean to depreciate. Similarly, the variance returns to zero for values ρ around 6.

### 4.2. Quantile Function

The quantile function is a measure that facilitates the simulation of random sample that first follows a uniform distribution, U(0,1), and then the distribution whose CDF is inverted to obtain the quantile function. Here, suppose u∼U(0,1) for X∼ DUS-TLE (ν,ρ); then, the quantile function is given as

$$x_{u}=-\frac{1}{2\rho }\ln\left\{1-{\left[\ln\left(1+(e-1)u\right)\right]}^{\frac{1}{\nu }}\right\}.$$

When u=0.5, we obtain the median lifetime of the distribution.

**Figure 5:**
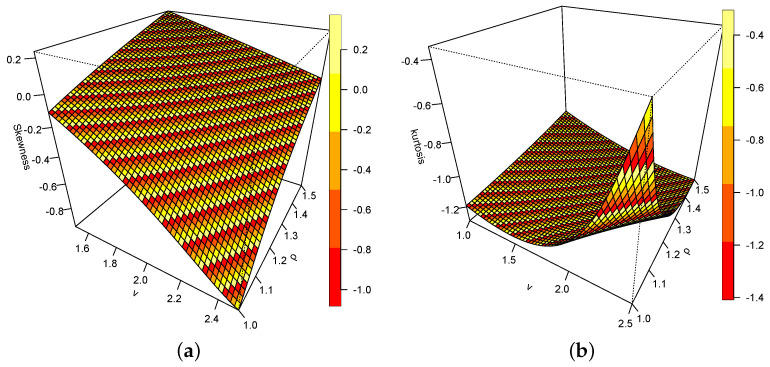
(**a**) Skewness of DUS-TLE. (**b**) Kurtosis of DUS-TLE.

**Figure 6:**
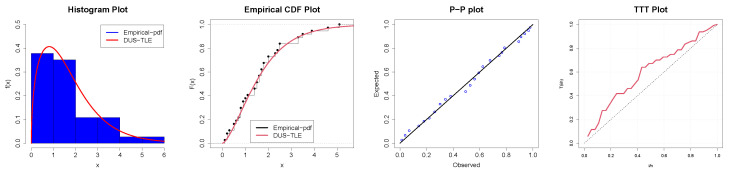
Parametric and non-parametric plots for Data-I.

Figure 5a,b are the plots of the skewness and kurtosis of the DUS-TLE distribution. The skewness plot suggests more of a negatively skewed model than positive. However, some values of the measure are positive, which rules out the possibility of only negative skewness. Therefore, there is visual evidence that the distribution can model both negatively and positively skewed data. From Figure 6, as ρ increases and ν reduces in value, the kurtosis reduces. Conversely, as ρ reduces and ν increases, the kurtosis increases.

### 4.3. Moment-Generating Function

The moment-generating function, $M_{X}\left(t\right)$, is a mathematical tool used to characterize the distribution of a random variable *X*. It is defined as the expected value of the exponential function of tX, i.e., $M_{X}\left(t\right)=E\left(e^{tX}\right).$ The $M_{X}\left(t\right)$ encapsulates all the moments of the distribution (e.g., mean, variance) and is useful in deriving properties of the random variable. For the random variable X∼ DUS-TLE (ν,ρ), the $M_{X}\left(t\right)$ is given as

MX(t)=E(etx)=2νρ∑i=0∞∑j=0∞∑k=0∞(−1)i+kν−1ijνk2ρ(1+k+i)−t−1.

where ν and ρ are parameters of the distribution.

### 4.4. Entropy

The Rény entropy by Rényi [27] is one of the popular measures of information loss. It is mathematically given as

Iα=11−αlog∫0∞fα(x;ξ)dx=11−αlog2νρe−1α∑i=0∞∑j=0∞∑k=0∞(−1)i+k2ρ(α+i+k)j!α(ν−1)iνjk,

where α≠1 and α>0.

### 4.5. Order Statistic

Given a random sample $X_{1},X_{2},\cdots ,X_{n}$ of size *n* from a population with CDF F(x;ν,ρ) and PDF f(x;ν,ρ), the *k*-th order statistic, $X_{\left(k\right)}$, represents the *k*-th smallest value in this sample. The PDF of the *k*-th order statistic $X_{\left(k\right)}$ from a sample of size *n* is generally given by

$$f_{X_{\left(k\right)}}\left(x;\nu ,\rho \right)=\frac{n!}{(k-1)!(n-k)!}{\left[F(x;\nu ,\rho )\right]}^{k-1}{\left[1-F(x;\nu ,\rho )\right]}^{n-k}f\left(x;\nu ,\rho \right).$$

By substituting F(x;ν,ρ) and f(x;ν,ρ) from Equations (7) and (8), the PDF of the *k*-th order statistic, $X_{\left(k\right)}$, for the ORET-L distribution is obtained as follows:

(12) $$\begin{array}{cc} f_{X_{\left(k\right)}}\left(x;\nu ,\rho \right) & =\frac{n!\cdot 2\nu \rho e^{-2\rho x}{\left[1-e^{-2\rho x}\right]}^{\nu -1}e^{{\left[1-e^{-2\rho x}\right]}^{\nu }}}{\left(k-1\right)!\left(n-k\right)!{\left(e-1\right)}^{k}}{\left(e^{{\left[1-e^{-2\rho x}\right]}^{\nu }}-1\right)}^{k-1}\\ \; & \times {\left(e-e^{{\left[1-e^{-2\rho x}\right]}^{\nu }}\right)}^{n-k}. \end{array}$$

Using the following general binomial and power series expansion identities,

ex=∑i=0∞eii!xi;(x−1)−n=∑j=0∞(−1)j+1n+j−1jxj,(x−1)n=∑k=0n(−1)knkxn−k,and(1−x)n=∑l=0∞(−1)lnlxl,

we can further decompose the expression in Equation (12) to become

fX(k)(x;ν,ρ)=n!2νρen−k(k−1)!(n−k)!(e−1)!∑i,j=0∞∑h,l=0∞∑m,n=0∞∑q,s=0∞∑t,w=0∞∑y=0∞∑r=0q∑z=0l−s(2ρ)qjnir(−1)i+j+h+q+t+w+y+l−s−zl!ν!n!q!hl−s−zkl−sν−1in−kjk−1hl−sz(l+m)νt(n−w)νy×nwxqe−2ρ(t+y)x.

## 5. Point Estimation

In this section, analytical estimation of the parameters of the DUS-TLE distribution is investigated using some non-Bayesian and Bayesian approaches. The essence of involving several methods is to determine their performance in large-sample scenarios.

### 5.1. Maximum Likelihood Estimation

Suppose we have a randomly selected group of *n* observations, represented as $x_{1},x_{2},\cdots ,x_{n}$, each drawn independently from the DUS-TLE distribution with unknown parameters ν and ρ. The likelihood and log-likelihood functions for the parameters ν and ρ take the following forms:

$$L\left(\nu ,\rho \right)=\prod_{i=1}^{n}f\left(x_{i},\nu ,\rho \right)=\prod_{i=1}^{n}\frac{2\nu \rho e^{-2\rho x}{\left[1-e^{-2\rho x}\right]}^{\nu -1}e^{{\left[1-e^{-2\rho x}\right]}^{\nu }}}{e-1}.$$

The log-likelihood is obtained as

(13) $$\begin{array}{cc} \ell (\nu ,\rho ) & =n\ln2+n\ln\nu +n\ln\rho -n\ln\left(e-1\right)-2\rho \sum_{i=1}^{n}x_{i}+\sum_{i=1}^{n}\ln{\left(1-e^{-2\rho x_{i}}\right)}^{\nu }\\ \; & +\left(\nu -1\right)\sum_{i=1}^{n}\ln\left(1-e^{-2\rho x_{i}}\right). \end{array}$$

Take the first partial derivative of Equation (13) with respect to ν:

(14) $$\frac{\partial \ell (\nu ,\rho )}{\partial \nu }=\frac{n}{\rho }+\sum_{i=1}^{n}{\left(1-e^{-2\rho x_{i}}\right)}^{\nu }\ln\left(1-e^{-2\rho x_{i}}\right)+\sum_{i=1}^{n}\ln\left(1-e^{-2\rho x_{i}}\right).$$

Take the first partial derivative of Equation (13) with respect to ρ:

(15) $$\frac{\partial \ell (\nu ,\rho )}{\partial \rho }=\frac{n}{\rho }-2\sum_{i=1}^{n}x_{i}+2\nu \sum_{i=1}^{n}x_{i}e^{-2\rho x_{i}}{\left(1-e^{-2\rho x_{i}}\right)}^{\nu -1}+2\left(\rho -1\right)\sum_{i=1}^{n}\frac{x_{i}e^{-2\rho x_{i}}}{1-e^{-2\rho x_{i}}}.$$

Equations (14) and (15) can be solved to find the MLE for ν and ρ using any numerical iteration algorithm.

### 5.2. Least Squares Estimation (LSE)

The method of Least Squares Estimation was introduced by Swain et al. [28] for determining the parameters of the Beta distribution. Building on the principles and findings presented by Swain et al. [28], we derive the following. Their work serves as a foundation for the approach used to estimate these parameters, which involves minimizing the sum of the squared differences between observed and theoretical values, ensuring the best possible fit of the Beta distribution model to the data. This technique is fundamental in deriving accurate estimates for the distribution’s parameters.

$$E\left[F\left(x_{j:n}|\nu ,\rho \right)\right]=\frac{j}{n+1};\;\mathrm{and}\;V\left[F\left(x_{j:n}|\nu ,\rho \right)\right]=\frac{j\left(n-j+1\right)}{{\left(n+1\right)}^{2}\left(n+2\right)}.$$

The parameters ν and ρ are estimated by the Least Squares method, yielding the estimates ${\hat{\nu }}_{LSE}$ and ${\hat{\rho }}_{LSE}$, which are found by minimizing the function L(ν,ρ) with respect to both ν and ρ.

(16) $$L\left(\nu ,\rho \right)=\arg\min_{\left(\nu ,\rho \right)}\sum_{j=1}^{n}{\left[F\left(x_{j:n}|\nu ,\rho \right)-\frac{j}{n+1}\right]}^{2}$$

The estimates are derived by solving the following set of non-linear equations:

(17) $$\sum_{j=1}^{n}{\left[F\left(x_{j:n}|\nu ,\rho \right)-\frac{j}{n+1}\right]}^{2}\Delta_{1}\left(x_{j:n}|\nu ,\rho \right)=0$$

(18) $$\sum_{j=1}^{n}{\left[F\left(x_{j:n}|\nu ,\rho \right)-\frac{j}{n+1}\right]}^{2}\Delta_{2}\left(x_{j:n}|\nu ,\rho \right)=0$$

where

(19) $$\Delta_{1}\left(x_{j:n}|\nu ,\rho \right)=e^{{\left(1-e^{-2\rho x}\right)}^{\nu }}{\left(1-e^{-2\rho x}\right)}^{\nu }\ln\left(1-e^{-2\rho x}\right),$$

and

(20) $$\Delta_{2}\left(x_{j:n}|\nu ,\rho \right)=2\nu xe^{{\left(1-e^{-2\rho x}\right)}^{\nu }-2\rho x}{\left(1-e^{-2\rho x}\right)}^{\nu -1}.$$

The results in Equations (19) and (20) are derived by taking the first partial derivatives of the CDF of the DUS-TLE distribution, presented in Equation (7), with respect to ν and ρ, respectively.

### 5.3. Weighted Least Squares Estimation (WLSE)

The parameters ν and ρ of the DUS-TLE distribution are estimated using the Weighted Least Squares method. The estimates, denoted as ${\hat{\nu }}_{WLSE}$ and ${\hat{\rho }}_{WLSE}$, are derived by minimizing the function W(ν,ρ) with respect to ν and ρ.

(21) $$W\left(\nu ,\rho \right)=\arg\min_{\left(\nu ,\rho \right)}\sum_{j=1}^{n}w_{j}{\left[F\left(x_{j:n}|\nu ,\rho \right)-\frac{j}{n+1}\right]}^{2}$$

where

$$w_{j}=\frac{{\left(n+1\right)}^{2}\left(n+2\right)}{j\left(n-j+1\right)}$$

(22) $$\sum_{j=1}^{n}w_{j}{\left[F\left(x_{j:n}|\nu ,\rho \right)-\frac{j}{n+1}\right]}^{2}\Delta_{1}\left(x_{j:n}|\nu ,\rho \right)=0$$

(23) $$\sum_{j=1}^{n}w_{j}{\left[F\left(x_{j:n}|\nu ,\rho \right)-\frac{j}{n+1}\right]}^{2}\Delta_{2}\left(x_{j:n}|\nu ,\rho \right)=0$$

where $\Delta_{1}\left(x_{j:n}|\nu ,\rho \right)$ and $\Delta_{2}\left(x_{j:n}|\nu ,\rho \right)$ are defined as outlined in Equations (19) and (20), respectively.

### 5.4. Maximum Product of Spacing Estimation (MPS)

Cheng and Amin [29] first introduced this method as an alternative to Maximum Likelihood estimation. The maximum product spacing technique offers a different approach, serving as an approximation to the Kullback–Leibler information criterion, rather than relying on the Maximum Likelihood method. Assuming the data are arranged in ascending order, we can proceed with this approach.

(24) $$I_{s}\left(data|\nu ,\rho \right)={\left[\prod_{j=1}^{n+1}D_{k}\left(x_{j:n}|\nu ,\rho \right)\right]}^{\frac{1}{n+1}}$$

where $D_{k}\left(x_{j:n}|\nu ,\rho \right)$ = $F\left(x_{j}|\nu ,\rho \right)$ - $F\left(x_{j-1}|\nu ,\rho \right)$; j=1,2,3,⋯,n. In a similar way, it is possible to opt for maximizing the function.

(25) $$N\left(\nu ,\rho \right)=\frac{1}{n+1}\sum_{j=1}^{n+1}\ln\left(D_{k}\left(x_{j:n}|\nu ,\rho \right)\right)$$

By differentiating the function N(ν,ρ) with respect to ν and ρ, and solving the resulting system of non-linear equations, $\frac{\partial N(\nu ,\rho )}{\partial \nu }=0$ and $\frac{\partial N(\nu ,\rho )}{\partial \rho }=0$, we can determine the parameter estimates.

### 5.5. Cramér–von Mises Estimation (CVME)

The parameters ν and ρ of the DUS-TLE distribution, denoted as the Cramér–von Mises estimates ${\hat{\nu }}_{CVME}$ and ${\hat{\rho }}_{CVME}$, are derived by minimizing the function C(ν,ρ) with respect to ν and ρ.

(26) $$C\left(\nu ,\rho \right)=\arg\min_{\left(\nu ,\rho \right)}\left\{\frac{1}{12n}+\sum_{j=1}^{n}{\left[F\left(x_{j:n}|\nu ,\rho \right)-\frac{2j-1}{2n}\right]}^{2}\right\}$$

The estimates are derived by solving the following set of non-linear equations:

(27) $$\sum_{j=1}^{n}\left[F\left(x_{j:n}|\nu ,\rho \right)-\frac{2j-1}{2n}\right]\Delta_{1}\left(x_{j:n}|\nu ,\rho \right)=0$$

(28) $$\sum_{j=1}^{n}\left[F\left(x_{j:n}|\nu ,\rho \right)-\frac{2j-1}{2n}\right]\Delta_{2}\left(x_{j:n}|\nu ,\rho \right)=0$$

where $\Delta_{1}\left(x_{j:n}|\nu ,\rho \right)$ and $\Delta_{2}\left(x_{j:n}|\nu ,\rho \right)$ are defined as outlined in Equations (19) and (20), respectively.

### 5.6. Anderson–Darling Estimation (ADE)

The Anderson–Darling estimators ${\hat{\nu }}_{ADE}$ and ${\hat{\rho }}_{ADE}$ for the parameters ν and ρ of the DUS-TLE distribution are derived by minimizing the function A(ν,ρ) with respect to both ν and ρ.

(29) $$A\left(\nu ,\rho \right)=\arg\min_{\left(\nu ,\rho \right)}\sum_{j=1}^{n}\left(2j-1\right)\left\{\ln\left(F\left(x_{j:n}|\nu ,\rho \right)\right)+\ln\left[1-F\left(x_{n+1-j:n}|\nu ,\rho \right)\right]\right\}$$

The estimates are derived by solving the following set of non-linear equations:

(30) $$\sum_{j=1}^{n}\left(2j-1\right)\left[\frac{\Delta_{1}\left(x_{j:n}|\nu ,\rho \right)}{F\left(x_{j:n}|\nu ,\rho \right)}-\frac{\Delta_{1}\left(x_{n+1-j:n}|\nu ,\rho \right)}{1-F\left(x_{n+1-j:n}|\nu ,\rho \right)}\right]=0$$

(31) $$\sum_{j=1}^{n}\left(2j-1\right)\left[\frac{\Delta_{2}\left(x_{j:n}|\nu ,\rho \right)}{F\left(x_{j:n}|\nu ,\rho \right)}-\frac{\Delta_{2}\left(x_{n+1-j:n}|\nu ,\rho \right)}{1-F\left(x_{n+1-j:n}|\nu ,\rho \right)}\right]=0$$

where $\Delta_{1}\left(x_{j:n}|\nu ,\rho \right)$ and $\Delta_{2}\left(x_{j:n}|\nu ,\rho \right)$ are defined as outlined in Equations (19) and (20), respectively.

### 5.7. Right-Tailed Anderson–Darling Estimation (RTADE)

The estimates ${\hat{\nu }}_{RTADE}$ and ${\hat{\rho }}_{RTADE}$ for the parameters ν and ρ of the DUS-TLE distribution, based on the Right-Tailed Anderson–Darling method, are determined by minimizing the function RA(ν,ρ) with respect to both ν and ρ.

(32) $$RA\left(\nu ,\rho \right)=\arg\min_{\left(\nu ,\rho \right)}\left\{\frac{n}{2}-2\sum_{j=1}^{n}F\left(x_{j:n}|\nu ,\rho \right)-\frac{1}{n}\sum_{j=1}^{n}\left(2j-1\right)\ln\left[1-F\left(x_{n+1-j:n}|\nu ,\rho \right)\right]\right\}$$

The estimates are derived by solving the following set of non-linear equations:

(33) $$-2\sum_{j=1}^{n}\frac{\Delta_{1}\left(x_{j:n}|\nu ,\rho \right)}{F\left(x_{j:n}|\nu ,\rho \right)}+\frac{1}{n}\sum_{j=1}^{n}\left(2j-1\right)\left[\frac{\Delta_{1}\left(x_{n+1-j:n}|\nu ,\rho \right)}{1-F\left(x_{n+1-j:n}|\nu ,\rho \right)}\right]=0$$

(34) $$-2\sum_{j=1}^{n}\frac{\Delta_{2}\left(x_{j:n}|\nu ,\rho \right)}{F\left(x_{j:n}|\nu ,\rho \right)}+\frac{1}{n}\sum_{j=1}^{n}\left(2j-1\right)\left[\frac{\Delta_{2}\left(x_{n+1-j:n}|\nu ,\rho \right)}{1-F\left(x_{n+1-j:n}|\nu ,\rho \right)}\right]=0$$

where $\Delta_{1}\left(x_{j:n}|\nu ,\rho \right)$ and $\Delta_{2}\left(x_{j:n}|\nu ,\rho \right)$ are defined as outlined in Equations (19) and (20), respectively. The estimates provided in Equations (14), (15), (17), (18), (22), (23), (25), (27), (28), (30), (31), (33) and (34) were derived using the *optim()* function in R, which implements the Newton–Raphson iterative method.

### 5.8. Bayesian Estimation

This section focuses on determining the Bayesian estimates (BEs) of the unknown parameters for the DUS-TLE distribution. In Bayesian parameter estimation, various loss functions can be utilized, such as the squared error, LINEX, and generalized entropy loss functions. To estimate the parameters, we can assume independent gamma priors for ν and ρ, with their probability density functions (PDFs) given as part of the prior distribution for the DUS-TLE model.

(35) $$\left.\begin{array}{ccc} \pi_{1}\left(\nu \right) & \propto & \nu^{p_{1}-1}e^{-q_{1}\nu },\;\nu >0,\;p_{1}>0,\;q_{1}>0\\ \pi_{2}\left(\rho \right) & \propto & \rho^{p_{2}-1}e^{-q_{2}\rho },\;\rho >0,\;p_{2}>0,\;q_{2}>0 \end{array}\right\}$$

The hyper-parameters $p_{j}$ and $q_{j}$, for j=1,2, are chosen based on prior knowledge regarding the unknown parameters. The joint prior distribution for ϕ=(ν,ρ) is defined as follows:

(36) $$\left.\begin{array}{cc} \pi_{1}\left(\phi \right) & =\pi_{1}\left(\nu \right)\pi_{2}\left(\rho \right)\\ \pi_{1}\left(\phi \right) & \propto \nu^{p_{1}-1}\rho^{p_{2}-1}e^{-q_{1}\nu -q_{2}\rho } \end{array}\right\}$$

The posterior probability distribution, conditioned on the observed data $X=(x_{1},x_{2},\cdots ,x_{n})$, can be expressed as follows:

$$\pi \left(\phi |X\right)=\frac{\pi (\phi )\iota (\phi )}{\int_{\phi }\pi \left(\phi \right)\iota \left(\phi \right)\;d\phi }$$

This suggests that the posterior density function is

(37) $$\pi \left(\phi |X\right)\propto \frac{2^{n}\nu^{p_{1}+n-1}\rho^{p_{2}+n-1}e^{-q_{1}\nu -q_{2}\rho -2\rho \sum_{i=1}^{n}x_{i}+\sum_{i=1}^{n}{\left[1-e^{-2\rho x_{i}}\right]}^{\nu }}}{{\left(e-1\right)}^{n}}\prod_{i=1}^{n}{\left[1-e^{-2\rho x_{i}}\right]}^{\nu -1}.$$

For any function τ(ϕ) and under the squared error loss (SEL) criterion, the Bayes estimator is expressed as follows:

(38) $${\hat{\phi }}_{BE\_SEL}=E\left[\tau \left(\phi \right)|x\right]=\int_{\phi }\tau \left(\phi \right)\pi \left(\phi |x\right)d\phi .$$

The SEL affects both underestimation and overestimation in the same way due to its asymmetric loss function. In numerous real-world scenarios, underestimating or overestimating can lead to significant consequences. In some cases, a LINEX loss function can be suggested as an alternative to the SEL, as described by

$$\left(\tau \left(\phi \right),\hat{\tau }\left(\phi \right)\right)=e^{\left\{\hat{\tau }\left(\phi \right)-\tau \left(\phi \right)\right\}}-\eta \left(\hat{\tau }\left(\phi \right)-\tau \left(\phi \right)\right)-1.$$

Here, η≠0 serves as a shape parameter. When η>1, it indicates that overestimations are considered more significant than underestimations, while η<0 suggests the opposite. As η approaches zero, the loss function converges to the standard squared error (SE) loss. For more information on this, one can consult Varian [30] and Doostparast et al. [31]. The Bayes estimator (BE) of τ(ϕ) under this loss is derived as follows:

(39) $${\hat{\phi }}_{\mathrm{BELINEX}}=E\left[e^{-\eta \;\tau (\phi )}\;|\;x\right]=-\frac{1}{\eta }\log\left(\int_{\phi }e^{-\eta \;\tau (\phi )}\;\pi \left(\phi |x\right)\;d\phi \right).$$

Furthermore, we incorporate the general entropy loss (GEL) function, as proposed by Calabria and Pulcini [32]. This function is characterized by the following definition.

$$\left(\tau \left(\phi \right),\hat{\tau }\left(\phi \right)\right)={\left(\frac{\hat{\tau }\left(\phi \right)}{\tau (\phi )}\right)}^{-l}-l\log\left(\frac{\hat{\tau }\left(\phi \right)}{\tau (\phi )}\right)-1.$$

The shape parameter l≠0 indicates a deviation from symmetry. When l>0, overestimations are considered more critical than underestimations, while for l<0, the reverse holds true. In this context, the Bayes estimator is presented with respect to the Generalized Error (GE) loss function;

(40) $${\hat{\phi }}_{\mathrm{BE}\_\mathrm{GEL}}=E{\left[{\left(\tau \left(\phi \right)\right)}^{-l}\;|\;x\right]}^{-1/l}={\left(\int_{\phi }{\left(\tau \left(\phi \right)\right)}^{-l}\pi \left(\phi |x\right)\;d\phi \right)}^{-1/l}.$$

The estimates produced by Equations (38), (39), and (40) cannot be simplified into closed-form solutions. Consequently, to generate posterior samples and obtain the corresponding Bayes estimates, the Markov chain Monte Carlo (MCMC) technique is utilized (see Brooks [33] & Van Ravenzwaaij et al. [34] for more details on the MCMC technique). In MCMC, an initial subset of random samples of size *M*, drawn from the posterior distribution, may be discarded as “burn-in.” The remaining samples are then employed to compute the Bayes estimates. Using MCMC within the framework of the SEL, LINEX, and GEL loss functions, the Bayes estimates (BEs) of $\phi^{\left(j\right)}=\left(\alpha^{\left(j\right)},\lambda^{\left(j\right)}\right)$ are calculated as follows:

(41) $${\hat{\phi }}_{\mathrm{BE}\_\mathrm{SEL}}=\frac{1}{M-\tau_{B}}\sum_{j=\tau_{B}}^{M}\phi \left(j\right),$$

(42) $${\hat{\phi }}_{\mathrm{BE}\_\mathrm{LINEX}}=-\frac{1}{\eta }\log\left(\frac{1}{M-\tau_{B}}\sum_{j=\tau_{B}}^{M}e^{-\eta \phi (j)}\right),$$

and

(43) $${\hat{\phi }}_{\mathrm{BE}\_\mathrm{GEL}}={\left(\frac{1}{M-\tau_{B}}\sum_{j=\tau_{B}}^{M}\phi {\left(j\right)}^{-l}\right)}^{-\frac{1}{l}},$$

where $\tau_{B}$ denotes the quantity of burn-in samples.

## 6. Simulation

In this study, several non-Bayesian methods were employed for comparison. These methods include Maximum Likelihood (ML), Maximum Product of Spacings (MPS), Least Squares (LS), Weighted Least Squares (WLS), Cramér–von Mises (CVM), Anderson–Darling (AD), and Right-Tailed Anderson–Darling (RTAD). For each of these methods, parameter estimates were computed for the 10,000 bootstrap samples across the four sample sizes. In addition to the non-Bayesian simulation, the Markov chain Monte Carlo (MCMC) technique was employed to generate posterior samples for Bayesian estimation due to the complexity of deriving closed-form solutions from Equations (37), (39), and (40). To further validate the accuracy of the proposed estimation methods, a bootstrap approach was used in conjunction with MCMC. Specifically, 10,000 bootstrap samples were generated for each sample size n=25,75,150,200. For each bootstrap sample, the MCMC algorithm was applied to estimate the parameters $\phi^{\left(j\right)}=\left(\nu^{\left(j\right)},\rho^{\left(j\right)}\right)$, with a portion of the initial samples discarded as “burn-in” to ensure convergence of the Markov chain. Using the remaining samples, Bayes estimates were computed under the frameworks of the squared error loss (SEL), LINEX loss, and general entropy loss (GEL) functions. To evaluate the performance of both Bayesian and non-Bayesian estimation methods, the average bias and root mean squared error (RMSE) were calculated for each sample size and across the 10,000 bootstrap iterations. These performance metrics provided a thorough assessment of the estimation accuracy. The results demonstrate how the Bayesian estimators, particularly when combined with MCMC, outperform the non-Bayesian methods in terms of reduced bias and lower RMSE, particularly as the sample size increases. By comparing the average bias and RMSE obtained across different sample sizes, the simulation study offers critical insights into the efficiency of the non-Bayesian and Bayesian estimation methods in various sample scenarios, highlighting the robustness of the MCMC approach for parameter estimation in complex distributions.

In interpreting the simulation results presented in Table 2, Table 3, Table 4 and Table 5, we observe the comparative performance of both non-Bayesian and Bayesian estimation methods in estimating the parameters ν and ρ of the DUS-TLE distribution across various sample sizes. For non-Bayesian methods like Maximum Likelihood (ML), Maximum Product Spacings (MPS), Least Squares (LS), Weighted Least Squares (WLS), Cramér–von Mises (CVM), Anderson–Darling (AD), and Right-Tailed Anderson–Darling (RTAD), the results show a trend of decreasing bias and RMSE values as sample size increases from n=25 to n=200. This suggests that these methods provide more accurate estimates as the sample size grows, with the ML method consistently yielding lower bias and RMSE values, indicating its robustness across different sample sizes.

On the other hand, the Bayesian estimation (BE) methods, such as BE with squared error loss (SEL), BE with Linex loss (Linex1 and Linex2), and BE with Generalized Entropy loss (GEL1 and GEL2), exhibit a different pattern. These methods generally show higher bias and RMSE compared to the non-Bayesian approaches, particularly at smaller sample sizes. Even though the Bayesian methods perform slightly better as the sample size increases, they do not match the efficiency of the non-Bayesian methods in terms of bias and RMSE reduction. Notably, the ${\mathrm{BE}}_{\mathrm{SEL}}$ method demonstrates the largest bias and RMSE across all sample sizes, suggesting that it may not be as reliable for the parameter estimation of DUS-TLE.

For both non-Bayesian and Bayesian methods, the estimation of ρ tends to have lower bias and RMSE compared to ν, implying that the parameter ρ is easier to estimate across all methods and sample sizes. Among all the methods, ML and LS perform well with lower bias and RMSE, especially as the sample size grows, while the Bayesian methods, despite improvements with larger sample sizes, remain less competitive overall. The general trend observed is that as the sample size increases, both bias and RMSE decrease for all methods. This highlights the importance of larger sample sizes in obtaining more reliable and accurate parameter estimates. However, even with larger sample sizes, non-Bayesian methods like ML and WLS continue to outperform the Bayesian approaches, particularly in terms of lower bias and RMSE for both ν and ρ.

Overall, the results indicate that non-Bayesian methods, particularly ML, consistently outperform Bayesian methods in terms of bias and RMSE when estimating the parameters ν and ρ, with performance improving as the sample size increases.

## 7. Applications

The first data type is the total factor productivity for thirty-seven African countries, extracted from https://documents.worldbank.org/en/publication/documents-reports/documentdetail/646931468157519398/total-factor-productivity-across-the-developing-world, accessed on 22 October 2024. The data is reported in Table 6. In Table 7, summary statistics providing preliminary insights into the data are presented. Observe that the skewness measure is 0.2, suggesting that the data are skewed to the right, and from theoretical visual evidence in Figure 5a, the DUS-TLE distribution can also model positively skewed data. Notice also that the kurtosis measure is 3.539668. This indicates highly peaked data (leptokurtic), and in Figure 5b, we show that our distribution can model highly peaked data. Consequently, in Table 8, the p-value of the DUS-TLE distribution is 0.9331, which is a near-perfect fit. Consider the p-value of common standard distributions such as gamma, log-normal, and Lomax; it is clear that the proposed DUS-TLE is superior in this study and hence can be extended to modeling related economic data. In Figure 6, we present the empirical density on a histogram of the Data-I, the CDF plot, P-P and TTT plots. These again provide supporting visual evidence that our model fits the TFP data well. In parameter estimating, the Lomax distribution is unusual as the estimates are too large for us to believe they emanated from the TFP data. The DUS-TLE model also has the lowest values of the model performance indicators, namely the log-likelihood (LL), Akaike Information Criterion (AIC), Consistent AIC (CAIC), Bayesian Information Criterion (BIC), Hannan–Quinn Information Criterion (HQIC), Cramér–von Mises (W), and Anderson–Darling (A) statistics.

**Figure 7:**
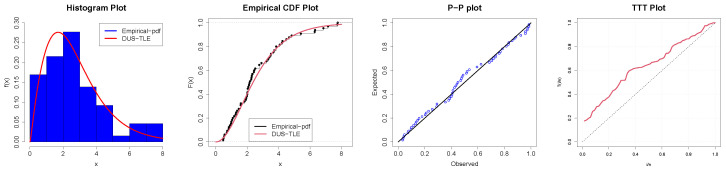
Parametric and non-parametric plots for Data-II.

The second application is on overall mortality rates among people who injected drugs studied by Mathers et al. [35] and presented in Table 9. In Table 10, the summary statistics are presented which reveal that the skewness measure is 0.02. This is also supported by the theoretical evidence in Figure 5a that the proposed distribution can fit right-skewed data. The kurtosis measure here is 3.689323, which implies leptokurtic data. In Table 11, the fitness measures for the distribution and its competitors are tabulated. The Kolmogorov–Smirnov (KS) statistic p-value for the DUS-TLE distribution is the highest, depicting a better fit to Data-II compared to the competing models. We further show some parametric and non-parametric plots in Figure 7.

## 8. Final Remarks

In this study, we introduced the DUS Topp–Leone family of distributions, characterized by its novel cumulative distribution function (CDF) and probability density function (PDF) defined in Equations (5) and (6). This new family of distributions enhances the traditional Topp–Leone framework by integrating the DUS transformer, thus offering a flexible modeling tool capable of capturing a range of lifetime behaviors. The derived DUS-TLE distribution, with its CDF and PDF represented in Equations (7) and (8), highlights the versatility of this approach in various contexts, from health outcomes to economic productivity. The behavior of the DUS-TLE distribution has been thoroughly examined, revealing a unique unimodal shape with properties such as a reversed bathtub shape and fat-tail characteristics. The hazard rate function (HRF) defined in Equation (9) supports this analysis, providing insights into the distribution’s applicability in modeling time-to-event data. Through graphical representations, we demonstrated the distribution’s bounded nature at both extremes, confirming its utility in scenarios where phenomena exhibit rises and falls in intensity. Parameter estimation using both non-Bayesian and Bayesian approaches further emphasizes the DUS-TLE distribution’s robustness. Our findings indicate that non-Bayesian methods, particularly Maximum Likelihood and Least Squares, consistently outperform Bayesian techniques in terms of bias and root mean square error (RMSE), especially as sample sizes increase. The comparison of estimation methods provides valuable insights into the reliability of the DUS-TLE distribution in practical applications. Additionally, we showcased the distribution’s capability to model datasets exhibiting varying skewness and kurtosis values, as evidenced by its fit to total factor productivity data across African countries and the mortality rate of people who injected drugs. The high p-value of the DUS-TLE distribution suggests an excellent fit compared to traditional distributions, reinforcing its potential as a preferred model for diverse applications in statistical analysis. Overall, the DUS Topp–Leone family, with its innovative features and robust performance, represents a significant advancement in statistical modeling, enabling researchers to better understand and interpret complex data patterns in various fields.

## Figures and Tables

**Table 1:** Some existing families of distributions.

Author	Family of Distributions
Marshall and Olkin [1]	Marshall-Olkin-G family of distributions
Eugene et al. [2]	Beta-generated family of distributions
Cordeiro and De Castro [3]	Kumaraswamy-G family of distributions
Alzaatreh et al. [4]	T-X generator of families of continuous distributions
Alzaghal et al. [5]	Exponentiated TX family of distributions
Aljarrah et al. [6]	T-X family of distributions using quantile function
Tahir et al. [7]	The logistic-X family of distributions
Al-Mofleh [8]	Family of distributions using Tangent function
Mahdavi and Kundu [9]	Alpha-Power transformed family of distributions
Gomes-Silva et al. [10]	The odd Lindley-G family of distributions
Ijaz et al. [11]	Gull Alpha Power family of distributions
Aldeni et al. [12]	Family of distributions from quantile of generalized lambda distribution
Yousof et al. [13]	Burr-Hatke-G family of distributions
Oramulu et al. [14]	Sine generalized family of distributions
Zhao et al. [15]	Type-I heavy-tailed family of distributions
Kumar et al. [16]	Dinesh-Umesh-Sanjay (DUS) transformer

**Table 2:** Average estimated bias and RMSE of non-Bayesian and Bayesian estimation methods, with DUS-TLE parameters at (n=25,75,150,200), ν=1.75 and ρ=2.75.

Method	Parameter	*n* = 25		*n* = 75		*n* = 150		*n* = 200	
		Bias	RMSE	Bias	RMSE	Bias	RMSE	Bias	RMSE
ML	ν	0.30680	0.79234	0.09219	0.15996	0.04751	0.06450	0.02241	0.04550
	ρ	0.19930	0.42719	0.07518	0.12277	0.02882	0.05810	0.01743	0.04210
MPS	ν	0.16516	0.38299	0.10045	0.12417	0.06373	0.05700	0.06527	0.04398
	ρ	0.22615	0.35714	0.10774	0.11675	0.07657	0.05963	0.06663	0.04421
LS	ν	0.09827	0.82764	0.02863	0.21277	0.00911	0.08767	0.00373	0.06727
	ρ	0.01115	0.50545	0.00177	0.16889	0.01296	0.07683	0.00500	0.05955
WLS	ν	0.11277	0.69385	0.04692	0.17505	0.02334	0.07285	0.01329	0.05418
	ρ	0.02160	0.44462	0.02784	0.13869	0.00389	0.06418	0.00673	0.04857
CVM	ν	0.39490	1.47194	0.11051	0.25323	0.04837	0.09549	0.03287	0.07151
	ρ	0.22219	0.64390	0.07630	0.18369	0.02377	0.07914	0.02256	0.06118
AD	ν	0.14738	0.60415	0.04846	0.16490	0.02249	0.07014	0.00990	0.05126
	ρ	0.06837	0.39784	0.03053	0.13086	0.00367	0.06305	0.00421	0.04702
RTAD	ν	0.31941	1.41797	0.09683	0.25231	0.04417	0.11288	0.02378	0.07481
	ρ	0.12260	0.53070	0.05212	0.15721	0.01211	0.07803	0.00970	0.05390
${\mathrm{BE}}_{\mathrm{SEL}}$	ν	0.55487	0.79907	0.78302	0.82006	0.98106	1.07951	1.04221	1.18535
	ρ	0.36408	0.43646	0.60924	0.47901	0.75776	0.62920	0.80136	0.68520
${\mathrm{BE}}_{\mathrm{Linex}1}$	ν	0.62844	0.96311	0.82644	0.90727	1.01226	1.14775	1.06848	1.24556
	ρ	0.41527	0.49233	0.63469	0.51296	0.77337	0.65376	0.81383	0.70580
${\mathrm{BE}}_{\mathrm{Linex}2}$	ν	0.48968	0.67696	0.74205	0.74333	0.95103	1.01621	1.01681	1.12884
	ρ	0.31524	0.39025	0.58422	0.44712	0.74229	0.60538	0.78898	0.66508
${\mathrm{BE}}_{\mathrm{GEL}1}$	ν	0.52801	0.75787	0.76711	0.79237	0.97011	1.05709	1.03317	1.16570
	ρ	0.34827	0.42386	0.60175	0.46978	0.75333	0.62247	0.79787	0.67957
${\mathrm{BE}}_{\mathrm{GEL}2}$	ν	0.47592	0.68470	0.73566	0.73951	0.94830	1.01332	1.01515	1.12717
	ρ	0.31677	0.40067	0.58674	0.45168	0.74446	0.60911	0.79087	0.66838

**Table 3:** Average estimated bias and RMSE of non-Bayesian and Bayesian estimation methods, with DUS-TLE parameters at (n=25,75,150,200), ν=2.75 and ρ=1.5.

Method	Parameter	*n* = 25		*n* = 75		*n* = 150		*n* = 200	
		Parameter	RMSE	Bias	RMSE	Bias	RMSE	Bias	RMSE
ML	ν	0.62385	3.27384	0.16483	0.42151	0.06410	0.18717	0.06424	0.13593
	ρ	0.09321	0.12497	0.03426	0.03025	0.01196	0.01452	0.01066	0.01049
MPS	ν	0.23316	1.38440	0.17644	0.31953	0.12937	0.17002	0.09199	0.12226
	ρ	0.11930	0.10781	0.05938	0.02991	0.04167	0.01533	0.03233	0.01093
LS	ν	0.33707	8.48090	0.05604	0.59520	0.00076	0.26796	0.01942	0.20502
	ρ	0.02555	0.15199	0.00156	0.04068	0.01050	0.02019	0.00488	0.01466
WLS	ν	0.36863	6.75922	0.08602	0.48424	0.02274	0.21730	0.03906	0.16493
	ρ	0.00221	0.13786	0.01092	0.03388	0.00130	0.01648	0.00225	0.01205
CVM	ν	0.95746	18.60741	0.20319	0.72522	0.06857	0.29164	0.07156	0.22052
	ρ	0.09027	0.18543	0.03629	0.04404	0.00809	0.02063	0.00908	0.01497
AD	ν	0.31447	2.74304	0.08520	0.44165	0.02010	0.20800	0.03527	0.15791
	ρ	0.01695	0.11474	0.01178	0.03182	0.00164	0.01601	0.00148	0.01178
RTAD	ν	0.88029	15.54110	0.15916	0.73757	0.07371	0.33997	0.06766	0.24252
	ρ	0.06513	0.16854	0.01888	0.04061	0.00597	0.02041	0.00552	0.01435
${\mathrm{BE}}_{\mathrm{SEL}}$	ν	0.67844	1.41534	1.16084	1.84049	1.58896	2.84247	1.73921	3.30766
	ρ	0.17403	0.09821	0.27674	0.10113	0.35621	0.13980	0.38250	0.15651
${\mathrm{BE}}_{\mathrm{Linex}1}$	ν	0.86690	1.98688	1.27584	2.19269	1.67748	3.16204	1.81700	3.61021
	ρ	0.18657	0.10422	0.28257	0.10459	0.35980	0.14243	0.38541	0.15879
${\mathrm{BE}}_{\mathrm{Linex}2}$	ν	0.52400	1.07366	1.05666	1.55771	1.50633	2.56306	1.66593	3.03775
	ρ	0.16176	0.09275	0.27095	0.09778	0.35264	0.13722	0.37960	0.15426
${\mathrm{BE}}_{\mathrm{GEL}1}$	ν	0.63330	1.32874	1.13397	1.77122	1.56968	2.77848	1.72276	3.24798
	ρ	0.16671	0.09550	0.27347	0.09932	0.35428	0.13843	0.38096	0.15533
${\mathrm{BE}}_{\mathrm{GEL}2}$	ν	0.54643	1.18074	1.08097	1.64005	1.53140	2.65406	1.69001	3.13116
	ρ	0.15213	0.09051	0.26693	0.09576	0.35042	0.13571	0.37787	0.15298

**Table 4:** Average estimated bias and RMSE of non-Bayesian and Bayesian estimation methods, with DUS-TLE parameters at (n=25,75,150,200), ν=2.5 and ρ=1.25.

Method	Parameter	*n* = 25		*n* = 75		*n* = 150		*n* = 200	
		Bias	RMSE	Bias	RMSE	Bias	RMSE	Bias	RMSE
ML	ν	0.56269	2.21375	0.14932	0.33317	0.06334	0.15198	0.06078	0.10010
	ρ	0.08839	0.08023	0.02728	0.02183	0.01380	0.01088	0.01137	0.00755
MPS	ν	0.20649	0.92956	0.15207	0.25429	0.10942	0.13591	0.07790	0.08988
	ρ	0.09463	0.06639	0.05121	0.02174	0.03174	0.01107	0.02492	0.00770
LS	ν	0.26941	2.59778	0.05353	0.49264	0.00398	0.21157	0.02736	0.15190
	ρ	0.00146	0.09369	0.00403	0.03038	0.00537	0.01459	0.00004	0.01061
WLS	ν	0.28144	2.09340	0.07386	0.38236	0.02694	0.17503	0.03967	0.11799
	ρ	0.01467	0.08091	0.00525	0.02481	0.00276	0.01216	0.00501	0.00858
CVM	ν	0.77818	5.01773	0.18445	0.60104	0.06550	0.23040	0.07385	0.16454
	ρ	0.10205	0.12013	0.02794	0.03274	0.01037	0.01502	0.01179	0.01095
AD	ν	0.32248	1.79021	0.07763	0.34288	0.02692	0.17012	0.03674	0.11330
	ρ	0.03265	0.07505	0.00757	0.02311	0.00300	0.01198	0.00433	0.00837
RTAD	ν	0.61784	4.30160	0.16501	0.61153	0.07456	0.26461	0.06682	0.17531
	ρ	0.05472	0.09869	0.01813	0.02927	0.00966	0.01436	0.00836	0.01012
${\mathrm{BE}}_{\mathrm{SEL}}$	ν	0.73799	1.42996	1.13777	1.72789	1.48489	2.47283	1.60244	2.80185
	ρ	0.18034	0.08540	0.25661	0.08445	0.31653	0.10983	0.33589	0.12038
${\mathrm{BE}}_{\mathrm{Linex}1}$	ν	0.89581	1.91333	1.23470	2.01506	1.55800	2.71767	1.66601	3.02873
	ρ	0.18951	0.08978	0.26092	0.08681	0.31918	0.11155	0.33803	0.12185
${\mathrm{BE}}_{\mathrm{Linex}2}$	ν	0.60603	1.11966	1.04915	1.49168	1.41613	2.25559	1.54223	2.59701
	ρ	0.17134	0.08132	0.25233	0.08215	0.31389	0.10813	0.33376	0.11893
${\mathrm{BE}}_{\mathrm{GEL}1}$	ν	0.69768	1.34820	1.11335	1.66632	1.46748	2.41898	1.58770	2.75259
	ρ	0.17407	0.08301	0.25376	0.08299	0.31485	0.10876	0.33455	0.11947
${\mathrm{BE}}_{\mathrm{GEL}2}$	ν	0.61987	1.20545	1.06514	1.54928	1.43289	2.31414	1.55837	2.65612
	ρ	0.16157	0.07853	0.24807	0.08011	0.31146	0.10664	0.33185	0.11767

**Table 5:** Average estimated bias and RMSE of non-Bayesian and Bayesian estimation methods, with DUS-TLE parameters at (n=25,75,150,200), ν=1.5 and ρ=2.0.

Method	Parameter	*n* = 25		*n* = 75		*n* = 150		*n* = 200	
		Bias	RMSE	Bias	RMSE	Bias	RMSE	Bias	RMSE
ML	ν	0.28923	0.68747	0.06090	0.09121	0.02797	0.04395	0.02971	0.03158
	ρ	0.15552	0.26020	0.04898	0.06401	0.02221	0.03398	0.01980	0.02398
MPS	ν	0.10818	0.32147	0.09584	0.07624	0.06335	0.04088	0.04342	0.02897
	ρ	0.15872	0.21058	0.08758	0.06293	0.05709	0.03460	0.04318	0.02425
LS	ν	0.15703	1.21881	0.01157	0.13360	0.00077	0.06268	0.01339	0.04779
	ρ	0.00977	0.30922	0.00269	0.08758	0.00524	0.04632	0.00210	0.03293
WLS	ν	0.16585	0.98256	0.02444	0.10774	0.01211	0.05068	0.02204	0.03891
	ρ	0.02039	0.27083	0.01369	0.07252	0.00736	0.03832	0.01135	0.02743
CVM	ν	0.41648	2.14639	0.07808	0.15745	0.03274	0.06761	0.03750	0.05116
	ρ	0.16217	0.38709	0.05317	0.09518	0.02228	0.04798	0.02276	0.03406
AD	ν	0.17118	0.63199	0.02501	0.09611	0.01150	0.04934	0.02031	0.03756
	ρ	0.05173	0.23968	0.01678	0.06785	0.00712	0.03757	0.01017	0.02686
RTAD	ν	0.39883	2.79388	0.06030	0.17341	0.02779	0.07340	0.03029	0.05555
	ρ	0.11914	0.33689	0.02793	0.08349	0.01324	0.04499	0.01298	0.03174
${\mathrm{BE}}_{\mathrm{SEL}}$	ν	0.58809	0.76387	0.72901	0.69061	0.85761	0.81980	0.89613	0.87370
	ρ	0.38965	0.35068	0.52297	0.33972	0.60835	0.40263	0.63299	0.42603
${\mathrm{BE}}_{\mathrm{Linex}1}$	ν	0.64248	0.88184	0.76112	0.74964	0.87973	0.86175	0.91461	0.90996
	ρ	0.41984	0.38310	0.53801	0.35681	0.61749	0.41416	0.64026	0.43551
${\mathrm{BE}}_{\mathrm{Linex}2}$	ν	0.53868	0.66966	0.69842	0.63746	0.83617	0.78035	0.87815	0.83929
	ρ	0.36052	0.32193	0.50812	0.32337	0.59927	0.39138	0.62575	0.41672
${\mathrm{BE}}_{\mathrm{GEL}1}$	ν	0.56585	0.72909	0.71558	0.66894	0.84857	0.80368	0.88869	0.85982
	ρ	0.37747	0.34041	0.51706	0.33346	0.60486	0.39837	0.63023	0.42253
${\mathrm{BE}}_{\mathrm{GEL}2}$	ν	0.52248	0.66583	0.68897	0.62740	0.83059	0.77217	0.87386	0.83257
	ρ	0.35319	0.32107	0.50523	0.32117	0.59787	0.38992	0.62472	0.41557

**Table 6:** Total factor productivity (TFP) for thirty-seven African countries from 2001 to 2010 (Data-I).

4.6	0.9	1.8	1.4	0.2	3.9	1.8	0.8	2.0	0.8	1.6	0.8	2.0
1.6	0.5	0.1	2.5	2.4	0.6	1.1	0.7	1.7	1.0	1.7	2.5	3.5
0.3	0.9	2.3	0.5	1.5	5.1	0.2	1.5	3.3	1.4	3.3		

**Table 7:** Summary statistics for Data-I.

$\bar{X}$	$S^{2}$	*S*	Min	Max	IQR	Sk	Ku	$S_{e}$	Range
1.677297	1.512492	1.229834	0.1	5.1	1.5	1.017765	3.539668	0.2	5

**Table 8:** MLEs, model performance, and goodness-of-fit measures using Data-I.

Dist	NLL	AIC	CAIC	BIC	HQIC	W	A	KS	*p*-Value	${\hat{\nu }}_{\mathrm{MLE}}$ (Shape)	${\hat{\rho }}_{\mathrm{MLE}}$ (Scale)
DUS-TLE	53.58	111.159	111.512	114.381	112.295	0.029	0.180	0.089	0.9331	1.5482	0.4679
TIHTE	54.0	111.992	112.345	115.214	113.128	0.032	0.194	0.108	0.7860	0.2857	2.6509
Gamma	53.66	111.337	111.690	114.559	112.473	0.033	0.204	0.092	0.9144	1.7613	0.9769
GIE	62.73	129.466	129.819	132.688	130.602	0.294	1.785	0.211	0.0731	1.2723	0.8900
Lnorm	56.19	116.701	117.054	119.923	117.837	0.104	0.659	0.112	0.7413	0.2970	0.9011
EIE	54.68	113.356	113.709	116.578	114.492	0.062	0.387	0.146	0.4101	0.2409	0.3679
Lomax	56.57	117.149	117.502	120.371	118.285	0.033	0.205	0.160	0.3026	20051134	11813144

**Table 9:** Overall mortality rates among people who injected drugs (Data-II).

2.01	6.32	3.52	2.15	5.42	2.04	2.77	2.26	1.95	1.00	2.45	0.74	0.98
1.27	2.77	3.68	1.18	1.09	1.60	0.57	3.33	0.91	7.14	2.08	3.85	1.99
7.76	2.52	1.47	4.67	4.22	1.92	1.59	4.08	2.02	0.84	6.85	2.18	2.04
1.05	2.91	1.37	2.43	2.28	3.74	1.30	1.59	1.83	3.85	6.30	4.83	0.50
3.40	2.33	4.25	3.49	2.12	0.83	0.54	3.23	4.50	0.71	0.48	2.30	7.73

**Table 10:** Summary statistics for Data-II.

$\bar{X}$	$S^{2}$	*S*	Min	Max	IQR	Sk	Ku	$S_{e}$	Range
2.724923	3.351263	1.830645	0.48	7.76	2.31	1.124585	3.689323	0.23	7.28

**Table 11:** MLEs, model performance, and goodness-of-fit measures using Data-II.

Distribution	LL	AIC	CAIC	BIC	HQIC	W	A	KS	*p*-Value	${\hat{\nu }}_{\mathrm{MLE}}$ (Shape)	${\hat{\rho }}_{\mathrm{MLE}}$ (Scale)
DUS-TLE	119.55	243.104	243.297	247.453	244.820	0.054	0.351	0.081	0.7835	2.2928	0.3518
TIHTE	121.68	246.150	246.344	250.499	247.866	0.142	0.473	0.113	0.3788	0.1172	3.7800
Weibull	120.43	244.861	245.055	249.210	246.577	0.081	0.532	0.095	0.5983	3.0561	1.5949
Lnorm	119.74	243.961	243.454	247.610	244.960	0.056	0.354	0.116	0.3416	0.7311	0.6950
Gumbel	121.8	247.599	247.793	251.948	249.315	0.078	0.531	0.088	0.6987	1.9259	1.2892

## Data Availability

This study utilized publicly available data, which are included in this paper.
